# Pannus formation after transcatheter aortic valve implantation resulting in prosthetic valve dysfunction: a case report

**DOI:** 10.1093/ehjcr/ytaf440

**Published:** 2025-09-23

**Authors:** Fabian Hundertmark, Anton Tomsic, Tamer Owais, Evaldas Girdauskas

**Affiliations:** Department of Cardiothoracic Surgery, University Hospital Augsburg, Stenglinstraße 2, 86152 Augsburg, Germany; Department of Cardiothoracic Surgery, University Hospital Augsburg, Stenglinstraße 2, 86152 Augsburg, Germany; Department of Cardiothoracic Surgery, Helios-University-Wuppertal, Arrenberger Straße 20, 42117 Wuppertal, Germany; Department of Cardiothoracic Surgery, University Hospital Augsburg, Stenglinstraße 2, 86152 Augsburg, Germany

**Keywords:** Subaortic pannus, Bioprosthetic valve dysfunction, Transcatheter aortic valve implantation, Sapien 3 aortic valve, Case report

## Abstract

**Background:**

Subaortic pannus formation (SAP) is a recognized complication following surgical aortic valve replacement (SAVR), typically manifesting around 5 years post-implantation. However, SAP occurrence after transcatheter aortic valve implantation (TAVI) remains poorly documented and investigated.

**Case summary:**

This case report presents a 79-year-old male who presented to our clinic with fatigue on exertion after having undergone TAVI with a 26-mm Edwards SAPIEN 3 valve 3 years prior to presentation. Echocardiography revealed elevated transprosthetic gradients, with no improvement after initiation of anticoagulation therapy. On cardiac tomography imaging, SAP was suspected. The patient underwent successful reoperative SAVR with concomitant mitral and tricuspid valve repair. Intraoperatively, severe pannus formation with subvalvular obstruction of the valve opening area was observed.

**Discussion:**

This case highlights the risk of early SAP formation following TAVI, underscoring the need for long-term follow-up and a more thorough understanding of the underlying pathophysiology.

Learning points

- Subaortic pannus formation (SAP) is a known complication following surgical aortic valve replacement, leading to bioprosthetic valve dysfunction (BVD). However, SAP can also occur after transcatheter aortic valve implantation, highlighting the need for awareness in this patient population.
- Cardiac imaging plays a crucial role in the assessment of BVD, as it enables detailed characterization of the underlying pathological mechanisms and guides clinical decision-making.
- Further research is needed to better understand the pathophysiology, prevention, diagnostic strategies, and therapeutic options for SAP in both surgical and transcatheter valve interventions.

## Introduction

Subaortic pannus formation (SAP) after surgical aortic valve replacement (SAVR) most often occurs 5 years after implantation, resulting in prosthetic valve dysfunction (PVD).^1^ The pathophysiologic mechanisms behind pannus formation are thought to involve the impact of turbulent transvalvular blood flow, a nonimmune inflammatory reaction against the prosthesis in the periannular neointima on the left ventricular septum, and increased shear stress.^2^ SAP is most frequently documented following SAVR, while data and case reports on SAP occurrence post-transcatheter valve replacement [transcatheter aortic valve implantation (TAVI)] remain limited. Herein, we present a case of a 79-year-old man who developed PVD due to SAP 3 years following his initial TAVI.

## Summary figure

**Figure:**
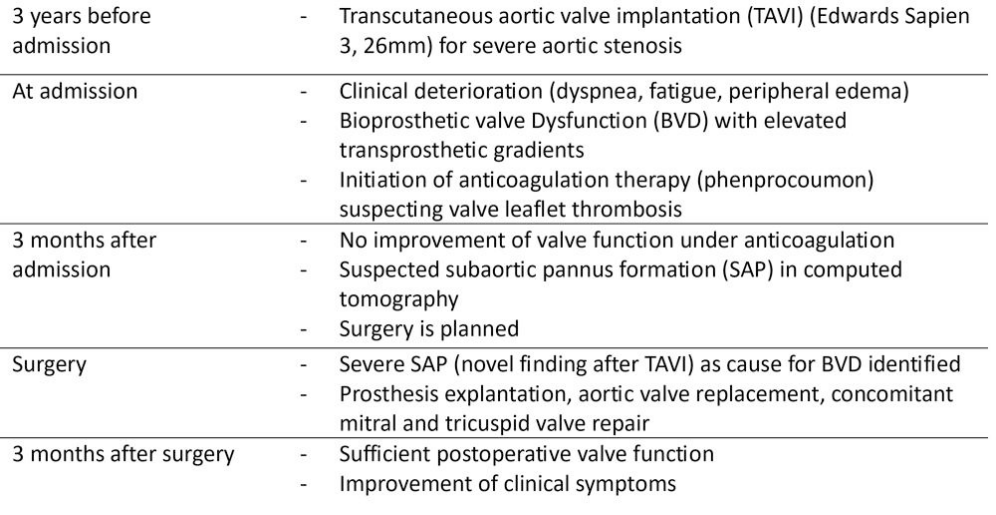


## Case presentation

**Figure 1:**
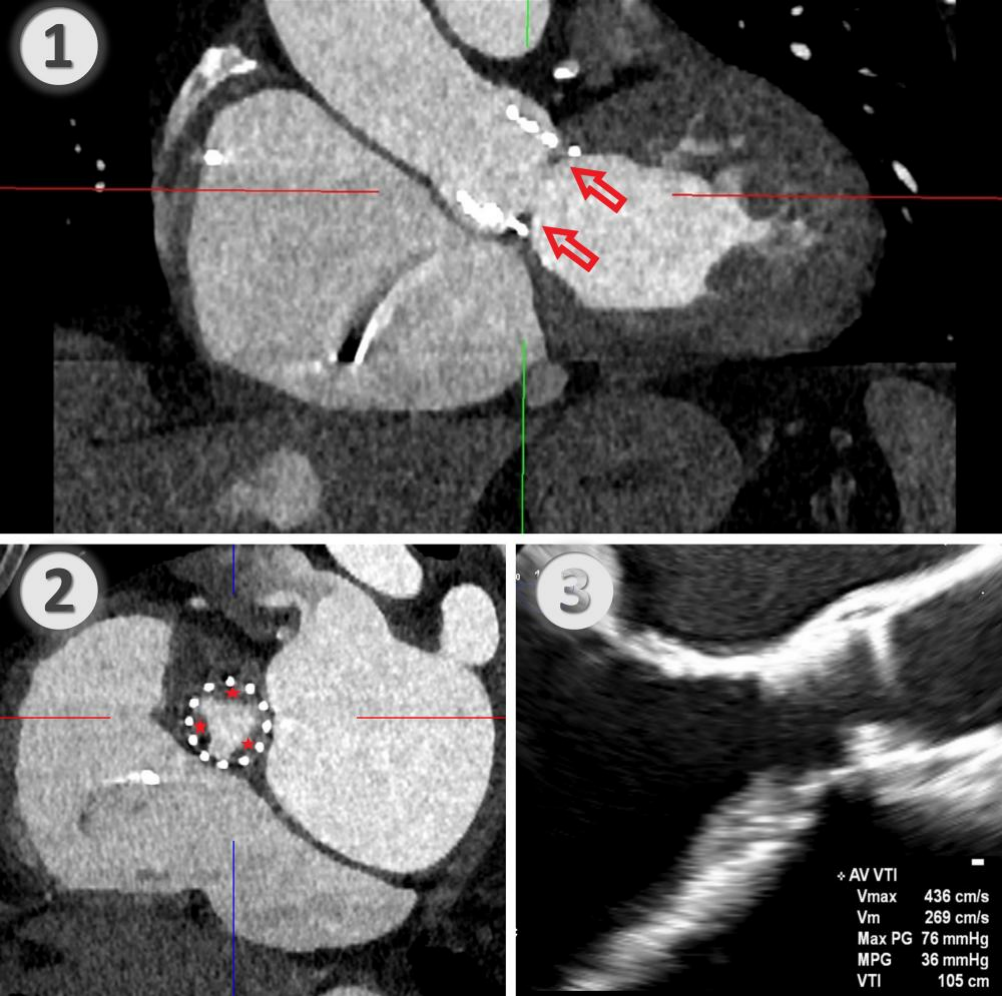
(*1*) Sagittal view of the prosthetic valve in contrast enhanced computed tomography. Arrowheads: pannus formation beneath valve leaflets. (*2*) Short axis view of prosthetic valve with pannus formation at the level of prosthetic valve cusps. Asterisks: pannus beneath all three valve leaflets. (*3*) Transoesophageal echocardiography images of the prosthetic valve in long axis view with transprosthetic gradients.

**Figure 2:**
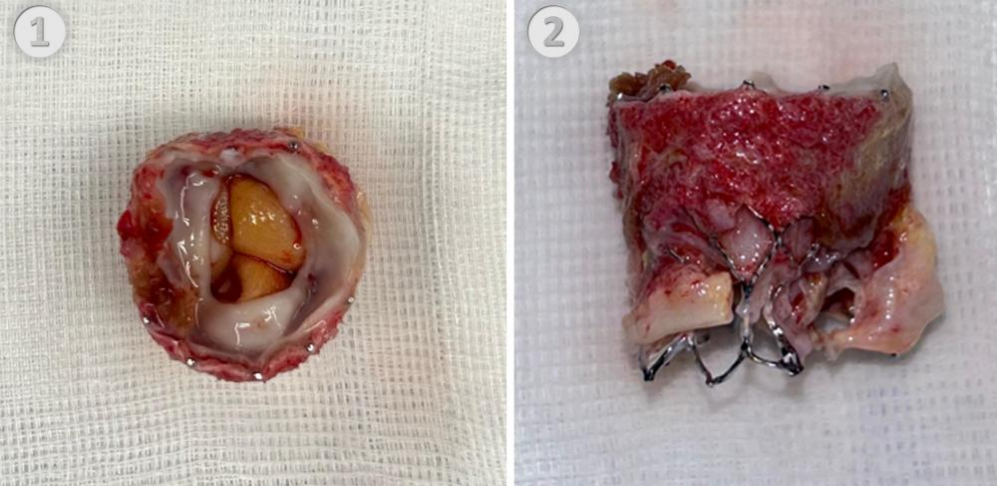
(*1*) Extracted bioprosthetic aortic valve (Edwards SAPIEN 3), with pannus formation on the ventricular inflow site. (*2*) Side view of the extracted bioprosthetic valve.

A 79-year-old man presented with progressive dyspnoea 3 years after undergoing transfemoral TAVI with a 26-mm Edwards SAPIEN 3 aortic valve (Edwards Lifesciences Corporation, Irvine, CA, USA) for severe aortic stenosis. Relevant comorbidities included chronic kidney failure, stable coronary artery disease, atrial fibrillation and permanent pacemaker implantation. Initially, a transprosthetic peak velocity of 1.9 m/s and a mean pressure gradient (PG) of 7 mmHg were documented. Three years after TAVI, echocardiography showed a transprosthetic peak velocity of 4.57 m/s and a mean PG of 44 mmHg as well as moderate mitral and tricuspid valve regurgitation. Laboratory testing showed elevated creatinine with 1.4 mg/dL and no other signs of organ dysfunction. Initial medical optimization included diuretics for cardiac recompensation and switching oral anticoagulation from apixaban to vitamin K antagonists for suspected prosthetic valve thrombosis. After 2 months, the patient failed to improve clinically, and echocardiography showed no improvement in aortic valve function. Computed tomography suggested SAP, showing a hypodense circular structure on the subannular inflow site of the aortic valve prosthesis (*Figure 1*). A SAVR through median sternotomy with concomitant mitral and tricuspid valve repair was scheduled. Intraoperatively, on the aortic side, the prosthesis appeared, largely unaltered with preserved leaflet mobility. On the ventricular side, however, extensive pannus formation was observed, obliterating the valve opening area, particularly under the right and non-coronary leaflets (*Figure 2*). The prosthetic valve was explanted while preserving the integrity of the aortic wall and aortic valve annulus. Mitral valve repair was achieved using a 30-mm IMR annuloplasty ring (Edwards Lifesciences Corporation, Irvine, CA, USA). A sutureless Perceval bioprosthesis Size L (CorCym, London, UK) was implanted in the aortic position, and tricuspid valve repair was achieved using a 32-mm Cosgrove ring (Edwards Lifesciences Corporation, Irvine, CA, USA). Postoperative echocardiography showed good results, with a transprosthetic aortic valve peak velocity of 3.0 m/s, a mean PG of 19 mmHg, and no paravalvular leakage.

## Discussion

SAP is a rare complication following heart valve replacement, with reported incidences of 0.3%, 5.0%, and 9.9% at 10, 20, and 25 years post-implantation.^3^ Risk factors for pannus formation include younger patient age, smaller prosthetic valve size, and multiple valve replacement.^3^ Diagnosing SAP is often challenging because transthoracic and transoesophageal echocardiography frequently indicate valvular dysfunction, such as aortic stenosis in our case, though often fail to elucidate the underlying pathomechanism when the valve leaflets are neither thickened nor calcified and retain adequate mobility. Differentiating pannus from thrombus is crucial, as thrombus formation may respond to anticoagulation, while pannus requires surgery. ESC/EACTS guidelines recommend cardiac CT for distinction, with pannus showing higher radiodensity [Hounsfield units (HU) > 145] compared to thrombus (HU > 90).^4^ The exact pathomechanism of SAP remains unclear, though increased immunologic activity is thought to contribute to accelerated valve degeneration and pannus formation.^5^ Other potential haemodynamic contributors include turbulent transvalvular flow and elevated wall shear stress, although these mechanisms have so far been described primarily in the context of surgically implanted prosthetic valves.^2^ To our knowledge, only one case following TAVI with a self-expanding valve has been described to date.^3^ No report of pannus formation after Edwards SAPIEN 3 aortic valve implantation is available in the literature. Compared to previous device generations, the latest generation of the SAPIEN 3 device includes a taller, textured polyethylene terephthalate outer skirt that successfully prevents paravalvular leakage post-implantation. This could lead to alterations in both immune activity and haemodynamic parameters, as described. As no reports on pannus formation with older-generation devices are available, the described pannus formation could be related to the design changes in the recent valve prosthesis generation. Currently, no data support this hypothesis, and it therefore remains primarily speculative. Clinical and imaging surveillance of the latest generation TAVI prostheses and their modifications seems warranted to determine whether our case presented an isolated finding, a more common occurrence, or even a clinically significant issue.

## Conclusion

While TAVI remains a viable option, particularly for high-risk surgical candidates, complications such as SAP may impact the long-term durability of transcatheter valves. Close monitoring of new valve devices seems warranted to better understand the clinical and echocardiographic prognosis associated with each specific model and its modifications.

## Lead author biography


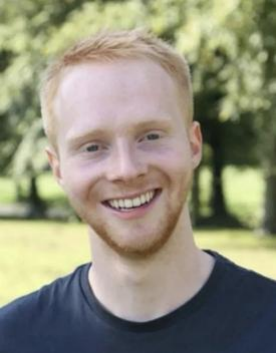


Fabian Hundertmark, MD, is a cardiac surgery resident in Augsburg, Germany, with a keen interest in advanced valvular interventions, structural heart disease, and aortic pathology. His research focuses on prosthetic valve dysfunction, as well as imaging techniques for structural and vascular diseases. Mr Hundertmark is committed to combining innovative surgical and diagnostic strategies with evidence-based medicine to enhance patient outcomes.

## Data Availability

Data sharing is not applicable to this article as all data relevant to the reported case have been presented in the article. Fabian Hundertmark (Conceptualization, Writing—original draft, Writing—review & editing, Visualization, Investigation), Anton Tomsic (Conceptualization, Writing—review and editing, Methodology), Tamer Owais (Resources), Evaldas Girdauskas (Conceptualization, Investigation, Resources, Supervision)
